# Editorial: Advances of novel approaches to enhance therapeutic efficacy and safety in human solid cold tumor

**DOI:** 10.3389/fimmu.2024.1398270

**Published:** 2024-03-22

**Authors:** Shicheng Guo, Xiaoting Xie, Yang Chen, Yanqing Liu, Lianxiang Luo

**Affiliations:** ^1^School of Life Sciences, Fudan University, Shanghai, China; ^2^The First Clinical College, Guangdong Medical University, Zhanjiang, Guangdong, China; ^3^Chinese Academy of Sciences (CAS) Key Laboratory of Separation Science for Analytical Chemistry, Dalian Institute of Chemical Physics, Chinese Academy of Sciences, Dalian, China; ^4^Herbert Irving Comprehensive Cancer Center, Columbia University, New York, NY, United States; ^5^The Marine Biomedical Research Institute, School of Ocean and Tropical Medicine, Guangdong Medical University, Zhanjiang, China

**Keywords:** cancer, immunotherapy, novel, efficacy, cold tumors, nanoparticle, epigenetic, biomarker

Tumor immunotherapy has garnered significant attention since its introduction in clinical practice. Unlike traditional treatments such as surgery, radiotherapy, and chemotherapy, immunotherapy offers the benefits of high specificity and minimal side effects. By stimulating the body to generate a targeted immune response against tumors, immunotherapy works to inhibit and destroy cancer cells. Immune checkpoint inhibitors, particularly CTLA-4 and PD-1/PD-L1 blockers, are crucial components of immunotherapy, as they help overcome the inhibitory signals that tumors send to immune cells, allowing for a more effective anti-tumor immune response (1, 2). Despite the promising potential of ICI in cancer therapy, a significant number of cancer patients exhibit resistance to this treatment. Additionally, the occurrence of immune-related adverse events (irAE) is not uncommon during ICI therapy, with severe cases potentially resulting in irreversible organ damage and life-threatening complications (3).

The effectiveness of immune checkpoint inhibitors (ICI) in treating cancer is influenced by various factors, including immune checkpoints, antigen presentation, the tumor microenvironment (TME), and inflammatory signals. “Cold tumors” like pancreatic cancer, colorectal cancer, and small cell lung cancer have low immunoreactivity, lack of effector T cells, high levels of immunosuppressive regulatory T cells and M2-polarized macrophages, limited antigen availability, and low T cell infiltration, making them less responsive to ICI treatment. Transforming “cold” tumors into “hot” tumors through strategies that enhance immunoreactivity could significantly improve the efficacy of immunotherapy (4). In this Research Topic, we focus on showcasing innovative approaches to enhance the effectiveness and safety of immunotherapy, discovering fresh biomarkers for “cold tumors”, enhancing results with cancer immunotherapy, and investigating methods to reshape the TME for promoting T-cell infiltration and immune reactions.

PD-1/PD-L1 inhibitors have shown remarkable therapeutic potential in clinical practice. Ni et al. found that, compared with EP/EC plus PD-L1 inhibitors, IP/IC combined with camrelizumab (a humanized high-affinity IgG4-κ anti-PD-1 monoclonal antibody) combined with apatinib has better efficacy and controllable safety in the treatment of untreated extensive-stage small-cell lung cancer (ES-SCLC). It was also found that the occurrence of irAE was associated with longer progression-free survival (PFS), which may be a potential prognostic factor for patients treated with ICIs. Sheng et al. used next-generation sequencing (NGS) for tumor gene expression profiling and PD-L1 immunohistochemical expression to achieve a precise diagnosis in a patient with primary cancer of unknown (CUP) who achieved complete remission after six cycles of nivolumab plus carboplatin and albumin-bound nanoparticle paclitaxel. In addition, Chen et al. innovated an attempt to use immunotherapy combined with etoposide-platinum (EP) regimen in the treatment of tracheal small cell carcinoma. The patient received six cycles of PD-L1 inhibitor (adebrelimab) combined with EP treatment, which significantly relieved symptoms and completely disappeared the tracheal mass. Wang et al. also tested that the addition of adebrelimab to etoposide-platinum-based chemotherapy may result in longer survival than durvalumab and atezolizumab in patients with ES-SCLC. Zhu et al. found that the strategic introduction of TACE and microwave ablation during PD-1 monoclonal therapy in patients with pancreatic cancer enhanced the release of tumor antigens, thereby amplifying the immune response. It not only shows the advantages of PD-1/PD-L1 inhibitors, but also implies the prospect of combination therapy in tumor immunotherapy. In addition to combination therapy, some emerging therapies, such as DNA/RNA vaccines, are also emerging. Liu et al. demonstrated that intramuscular injection of OVA-encoding plasmid DNA (pDNA) in combination with pDNA encoding α-PD-1 could enhance tumor therapy through *in situ* gene delivery and enhancement of a potent muscle-specific promoter. Similarly, in refractory brain tumors, antigen-specific personalized mRNA vaccines have shown effective anti-tumor responses in clinical brain tumor models, providing new ideas for therapeutic approaches targeting classical immunotherapy resistance and some “cold tumor” types (5). Muraro et al. also found that targeting BRAF mutations in cutaneous melanoma (CM), using an immune weapon as antibody-dependent cytotoxicity (ADCC) of anti-receptor tyrosine kinase (RTKs) antibodies, it effectively and specifically killed EGFR-expressing BRAFi resistant CM cells both *in vitro* and *in vivo* in a humanized mouse model.

Recent research has identified new biomarkers for “cold tumors” beyond the traditional PD-1/PD-L1 and CTLA-4 pathways. These biomarkers include surface proteins and non-coding RNA/DNA, offering new avenues for treating “cold tumors”. Lodewijk et al. have demonstrated that post-translational modifications on tumor cells can create tumor-associated antigens (TAAs), which are specific targets that can provoke anti-tumor immune responses. They have highlighted three TAAs: CD44v6, truncated carbohydrates like Tn and STn, and altered ganglioside expression such as GD2 and O-GD2. These TAAs show promise for developing antibodies, antibody-drug conjugates, nanodrugs, vaccines, and CAR T cell therapy. Additionally, researchers have observed that the escape of the prostate-specific antigen STEAP1 can lead to drug resistance in metastatic prostate cancer. Combining tumor-targeted interleukin-12 therapy with STEAP1-directed CAR T cell therapy has shown potential in overcoming STEAP1 antigen escape and enhancing the anti-tumor effects in prostate cancer (6). In addition to targeting surface proteins, certain researchers have employed an exosome-mediated CRISPR/Cas9 delivery system to target the YTHDF1 gene *in vivo*. This approach can effectively inhibit the translation of lysosomal genes, thereby restricting the lysosomal proteolysis of major histocompatibility complex class I (MHC-I) and antigens. Ultimately, this method helps to restore tumor immune surveillance (7). Likewise, certain researchers have employed mRNA lipid nanoparticles encoding just the N terminus of gasdermin to stimulate pyroptosis, resulting in a robust anti-tumor immune response and rendering “cold tumors” susceptible to checkpoint immunotherapy (8). By increasing the amount of cytoplasmic double-stranded DNA to activate the classical cGAS-STING-pTBK1/pIRF3 axis, IR and ATRi can enhance CD8+ T cell infiltration and improve the efficacy of anti-PD-L1 therapy in a mouse CRC model (9).

Remodeling the TME is a viable option for enhancing the immune reactivity of “cold tumors”. In a study by Hu et al., targeting neutrophils and combining various doses of radiation effectively inhibited tumor growth, disrupted the tumor cell cycle, and facilitated the release of neoantigens from tumor cells to enhance the immune response and prolong the cytotoxic effects of functional T cells. Additionally, some researchers have demonstrated that using nanomedicines to target different inhibitory immune cells can help minimize the side effects of drugs (10). By utilizing single-cell RNA sequencing (scRNA-seq), researchers can conduct a detailed analysis of the specific immune cell populations, their phenotypes, and other microenvironmental elements in the tumor microenvironment (TME) of different cancer types. This approach offers a more accurate method for tailoring immunotherapy treatments using immune checkpoint inhibitors (ICIs) (11). In a similar vein, Hapuarachi et al. propose that exercise training could potentially convert a “cold tumor” to a “hot tumor,” resulting in alterations to the tumor microenvironment (TME) and systemic immune system that could improve the efficacy of immunotherapy. Moreover, the administration of platinum drugs may trigger the release of cytokines and provoke varied responses within the TME, ultimately causing type I hypersensitivity that poses a challenge to patient treatment. To address this issue, Li and Yin suggest a 5-step platinum desensitization protocol to reduce the risk of allergic reactions.

This Research Topic explores novel approaches to enhance the effectiveness and safety of immunotherapy for “cold tumors” as well as underscores the dynamic field of tumor immunotherapy, highlighting the shift towards personalized and more targeted approaches to overcome the challenges associated with “cold tumors” and improve patient outcomes. These strategies include the identification of new biomarkers for “cold tumors”, targeting immune cells to modify the tumor microenvironment, and utilizing combination therapy to enhance the outcomes of cancer immunotherapy. These approaches offer a fresh perspective on the application of immunotherapy in treating “cold tumors”. While the potential for these strategies is promising, there are significant challenges in translating them into clinical practice. The discovery of new targets is eagerly anticipated, as they may offer additional avenues for improving tumor immunotherapy and advancing cancer prevention, diagnosis, and treatment.

## Author contributions

SG: Supervision, Writing – original draft, Writing – review & editing. XX: Writing – original draft. YC: Writing – review & editing. YL: Writing – review & editing. LL: Conceptualization, Writing – review & editing.
